# Clinical and epidemiological aspects of a hepatitis E outbreak in Bangui, Central African Republic

**DOI:** 10.1186/1471-2334-11-93

**Published:** 2011-04-14

**Authors:** Alice I Goumba, Xavier Konamna, Narcisse P Komas

**Affiliations:** 1 Viral Hepatitis Laboratory, Institut Pasteur de Bangui, PO Box 923, Bangui, Central African Republic

## Abstract

**Background:**

Outbreaks of hepatitis E frequently occur in tropical developing countries during the rainy season due to overflowing drains, short-circuiting of networks of clean water and use of contaminated water from wells. Hepatitis E virus (HEV) infections are usually accompanied by general symptoms of acute liver disease. This study was conducted to define the clinical and epidemiological aspects of the HEV outbreak that occurred in May 2004 in Bangui.

**Methods:**

Blood samples were collected from 411 patients aged 1-87 years, most of whom presented with jaundice, asthenia or signs of uncomplicated malaria, for a transversal study from June 2004 to September 2005. Patients were recruited at 11 health care centres, including two referral hospitals, after they had given informed consent. The diagnosis of HEV was made with a commercial ELISA test to detect IgM and/or IgG antibodies. HEV RNA was amplified by RT-PCR to confirm the presence of the viral genome.

**Results:**

The most frequent clinical signs found were jaundice (93.4%), vomiting (50.7%), hepatalgia (47.4%), hepatomegaly (30.9%) and asthenia (26.8%), which are the general clinical signs of hepatic disease. Acute hepatitis E was found in 213 patients (51.8%) who were positive for HEV IgM antibodies. The IgG anti-HEV seroprevalence during this outbreak was high (79.5%). The age group 18-34 years was more frequently infected (91.2%) than those aged 1-17 (78.0%) or over 34 (64.9%) (p < 10^-6^). RT-PCR performed on 127 sera from the 213 IgM-HEV-positive patients was amplified, and the presence of the viral genome was found in 65 samples.

**Conclusion:**

Although no specific clinical signs exist for hepatitis E infection, people presenting with jaundice, vomiting, hepatalgia, asthenia, hepatomegaly or distended abdomen with no signs of uncomplicated malaria in tropical developing countries should be sent to a laboratory for testing for hepatitis E.

## Background

Hepatitis E virus (HEV), a small, single-stranded, hepatotropic RNA virus, currently classified in the genus *Hepevirus *[1], is responsible for enteric non-A non-B hepatitis in humans. Infection with HEV, thought to spread via the faecal-oral route, causes outbreaks that have been linked to waterborne sources in developing countries; sporadic cases have also been seen [2]. Although the potential for zoonotic and cross-species transmission has been demonstrated [3,4], asymptomatic carriers of HEV have been reported, and such cases are potential human reservoirs of the virus [5]. During epidemics, the person-to-person transmission rate appears to be low, although intrafamilial transmission is possible [6]. The course of the disease is generally self-limiting; however, chronic HEV infection has been reported in immunocompromised patients [7-10]. Reports have indicated an increased risk for disease and as high as 10-20% case mortality from fulminant hepatitis during the third trimester of pregnancy [11].

Outbreaks of hepatitis E frequently occur in tropical Africa during the rainy season due to overflowing drains, short-circuiting of networks of clean water and use of contaminated water from wells [12-14]. The Central African Republic (CAR), located in tropical Africa, is considered to be an area of high endemicity for the main infectious diseases, including infections with HIV [15], hepatitis B virus and other hepatotropic viruses [16,17], yellow fever [18], malaria [19], tuberculosis [20] and other infections [21-23]. No HEV epidemics were documented in the CAR before 2001, although HEV antibodies were detected in 24% of young sexually active adults [24]. The first outbreak of this disease in the CAR was reported in 2001 [25,26], and a further outbreak occurred in 2004.

In order to improve the diagnosis of hepatitis E in CAR, the aim of this study was to determine the clinical and epidemiological characteristics of HEV infection during the outbreak in Bangui.

## Methods

### Study population

One month after the beginning of the 2004 outbreak, 411 patients residing in or around Bangui, aged 1-87 years (average age, 27.9 ± 5.1), were clinically examined by physicians in 11 health care centres, including two national referral hospitals, and completed a questionnaire to provide sociodemographic information, including gender, age and place of residence (district in Bangui). During standard clinical screening, all persons enrolled in the study were also questioned about their history of symptomatic hepatitis. The main criteria for inclusion in the study were gastrointestinal complaints and fever, leading to a clinical diagnosis of malaria with ineffective malaria treatment. Other inclusion criteria were jaundice, anorexia, diarrhoea, nausea or severe asthenia. Individuals should also have had no history of exposure to blood, such as transfusion.

Informed consent was obtained from all patients included in the study. For those under 18, parental consent was obtained. Each participant or parent was informed of the results of the serology. The study was approved by the Medical Committee of the Institut Pasteur in Paris (France) because of, until the third trimester of 2006, the Central African Republic did not have his own Ethics Committee. Before that time, all the research projects at the Institut Pasteur de Bangui (which is related to Institut Pasteur in Paris, France) were submitted to the Ethics Committee of the Institut Pasteur in Paris (France). Once ethical approval was received, administrative authorization was obtained from the Ministry of the Public Health of the Central African Republic.

### Clinical observations

Patients were examined for jaundice, hepatalgia, vomiting, asthenia, fever, arthralgia, abdominal pain, nausea, anorexia, diarrhoea or headache. They were then examined physically for hepatomegaly, distended abdomen, splenomegaly, oedema of lower limbs, encephalopathy or deterioration of general condition. Physicians could also report any clinical observation not listed in the questionnaire.

### Specimen collection

Blood and stool samples were collected from patients who fulfilled the inclusion criteria over 16 months between June 2004 and September 2005.

### Biological testing

A diagnosis of acute hepatitis E was based on serology. IgM and IgG HEV were detected with a commercial ELISA assay (Bioelisa, Biokit, Spain). Amplification of HEV RNA by RT-PCR was performed to confirm a recent HEV infection. Serum samples were also analysed for AST and ALT. Malaria, hepatitis B and C, and yellow fever were ruled out.

### Statistical analysis

Epi-Info Version 2000 (CDC, USA & WHO, Geneva, Switzerland) software was used to analyse data on prevalence, with 95% confidence intervals, and the chi-square test was used for the comparison of variables and associations with HEV positivity. Statistical significance was assumed at *p *< 0.05.

## Results

The sex ratio was of 1 (1:1) with 50.9% males (209) and 49.1% females (202). Serology results showed that more males were infected during the outbreak than females. IgM anti-HEV antibodies, which predominate in the primary immune response and define acute infection, were detected in the sera of 118 men (56.4%) and 95 women (47.0%). The observed difference was not significant. However, males had a higher IgG anti-HEV seroprevalence (85.1%) than females (73.8%), and the risk for infection was clearly higher in males than females (OR 2.04; 95% CI 1.21-3.45; *p *< 0.005) during the period of study.

Table 1 shows the distribution of HEV serology (IgM and IgG) according to age. The age group 18-34 years were more frequently IgM-HEV positive than the other age groups (1-17 and > 34 years) (*p *< 0.001).

**Table 1:** Distribution of 411 patients according to serology during the outbreak of hepatitis E in Bangui in 2004

Age (years)	IgM positive (%)	IgG positive (%)	Total
1-17	28 (56.0)	39 (78.0)	50 (12.2%)
18-34	126 (61.7)	186 (91.2)	204 (49.6%)
> 34	59 (37.6)	102 (64.9)	157 (38.2%)
Total	213 (51.8%)	327 (79.5%)	411

Amplification by RT-PCR of 127 serum and 25 stool samples positive for IgM-HEV antibody showed that 50 sera and 24 stool samples were positive, indicating that the viral genome was present.

More than 80% of patients with positive IgM anti-HEV antibody had higher ALT (82.1%) and AST (86.8%) values than normal.

The clinical features observed in patients positive for IgM-HEV are listed in Table 2. Although no specific symptoms were reported during the outbreak, jaundice was observed in most patients (93.4%), followed by vomiting (50.7%), hepatalgia (47.4%), asthenia (26.8%) and physical signs like hepatomegaly (30.9%) and distended abdomen (14.5%).

**Table 2:** Clinical features in 411 patients clinically examined during the outbreak of hepatitis E in Bangui in 2004

Clinical characteristics	Positive IgM-HEV (n = 213)
*Clinical signs*	

Jaundice (n = 353)	199 (93.4%)
Vomiting (n = 213)	108 (50.7%)
Hepatalgia (n = 212)	101 (47.4%)
Hepatomegaly (n = 142)	66 (30.9%)
Fever (n = 56)	31 (14.5%)
Abdominal pain (n = 18)	9 (4.2%)
Dark urine (n = 16)	8 (3.7%)
Nausea (n = 11)	7 (3.3%)
Itching (n = 9)	6 (2.8%)
Encephalopathy (n = 10)	5 (2.3%)
Myalgia (n = 3)	3 (1.4%)
Hemorrhage (n = 6)	1 (0.5%)
Discolored stools (n = 4)	1 (0.5%)

*Clinical symptoms*	

Asthenia (n = 84)	57 (26.8%)
Distended abdomen (n = 64)	31 (14.5%)
Epigastralgia (n = 19)	12 (5.6%)
Arthralgia (n = 19)	11 (5.2%)
Anorexia (n = 10)	8 (3.7%)
Limb oedema (n = 25)	7 (3.3%)
Headache (n = 10)	4 (1.9%)
Diarrhoea (n = 7)	4 (1.9%)
Ascites (n = 5)	3 (1.4%)
Splenomegaly (n = 5)	1 (0.5%)
Deterioration of general condition (n = 5)	0
Cutaneous eruption (n = 1)	0

Percentages of total of positive IgM (213)

**Figure 1:**
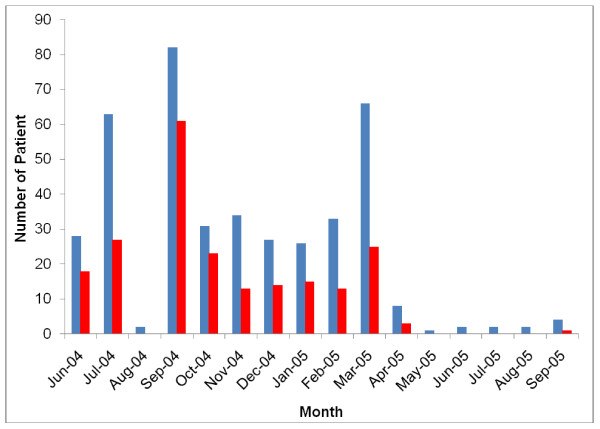
**Evolution of IgM-HEV serology during 16 months, between June 2004 and the beginning of the outbreak to September 2005**. Blue represents the total numbers of patients; red shows the number with detected IgM-HEV.

To determine how long the infection persisted in the population, variations in IgM HEV positivity were monitored for 16 months, from June 2004, 1 month after the beginning of the outbreak, to September 2005 (Figure 1). The number of cases with IgM-HEV peaked in July 2004, September 2004 and March 2005. Between October 2004 and February 2005, between the two outbreaks, the level of infection remained constant at the background level.

**Figure 2:**
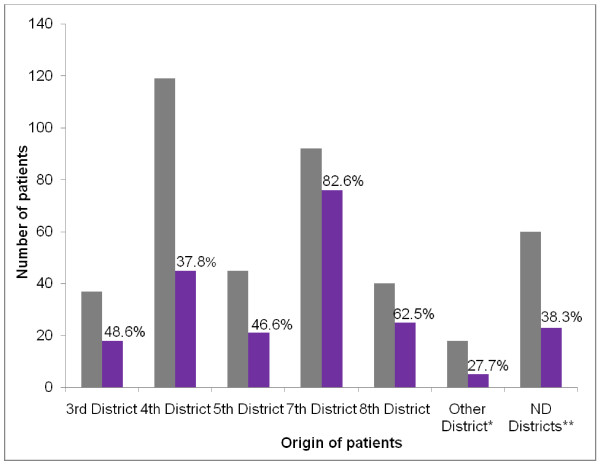
**Locations of patients with positive IgM-HEV in Bangui and suburbs**. Black represents the total number of patients; purple shows the number of patients who were IgM-HEV antibody-positive. Patients were from all eight districts of Bangui. *Other districts: first, second and third districts **ND district: district not determined

In order to define the HEV-infected area, we collected blood and stools in different health care centres in the eight districts of Bangui, each of which has at least one public health care centre. As shown in Figure 2, IgM-HEV was detected in patients from almost all districts of Bangui; however, more patients in the fourth and seventh districts presented for medical examination, and higher percentages of HEV infection were found in the seventh and eighth districts.

## Discussion

During the 2004 outbreak of hepatitis E, more than 51% of patients were infected with HEV. The most frequent clinical and biological signs were jaundice, vomiting, hepatalgia, hepatomegaly, asthenia, distended abdomen, fever and high levels of transaminases. A possible limitation of this study is the wide inclusion criteria, as physicians at the health care centres could include all patients who presented with jaundice or uncomplicated malaria. It is possible that this inclusion criterion biased our study, because other patients might not have been included or did not enter the defined categories. Nevertheless, the rate of IgM-HEV obtained was very high and was in accordance with that previously reported; e.g. 42% of patients were infected at the time of an epidemic of hepatitis E in Namibia [14].

The high prevalence of jaundice among our patients may be explained by the fact that CAR patients are more aware of this sign, because it is usually related to yellow fever and thus requires urgent medical examination. A study conducted in Pakistan [27] also showed that jaundice was present in almost all patients with HEV IgM positive serology. This sign was also found in 10 IgM-HEV-positive patients in Nigeria [28]. Our study shows, however, that the presence of jaundice cannot confirm HEV infection, and vomiting, hepatalgia, asthenia, fever, elevated ALT levels and hepatomegaly, distended abdomen and fever must also be present [29]. As previously reported [30], we also found that adolescents and young adults were preferentially infected by HEV during the epidemic. This observation suggests that active people are more readily in contact with the virus, especially in tropical developing countries. In these countries, many people are farmers, who may be a source of contamination by HEV, because poultry and swine farmers, other professionals with an occupational activity related to farming and veterinarians are occupational risk groups and are more heavily exposed to HEV than other professions [31-35].

Biochemical determination of hepatic cytolysis by the detection of high levels of transaminases is an important parameter in the control of liver disease. In our study, the level of transaminases was four to five times higher than normal in more than three quarters of the infected patients. This confirms the presence of hepatic cytolysis, which usually accompanies infection with hepatitis viruses [36]. RT-PCR was not positive in all cases with IgM HEV, confirming that detection of the viral genome is generally difficult after the beginning of symptoms during epidemics of hepatitis E [37]. Our results could not formally link HEV infection to transaminases, because of the prevalence of many other infectious diseases, including other types of hepatitis, yellow fever and malaria, which may also increase hepatic enzymes [38].

The main inclusions occurred in July and September 2004 and March 2005, which correspond to the peaks of infection and were linked to positive IgM-HEV serology. In Bangui, the rainy season starts in May and finishes in December, and a recrudescence of cases of acute hepatitis E is observed during that period in tropical countries, due to overflowing drains and short-circuiting of networks of clean water and structures for purifying wastewater. The level of endemicity results in high IgG anti-HEV seroprevalences. As there is generally a single source of contamination, generally from water, epidemics of hepatitis E are characterized by spectacular numbers of infected people [37,39,40]. The lack of samples during August was probably related to the absence of health professionals, who take their annual vacation at this time. The outbreak may therefore have peaked during August, and the peak observed in September might be only the downward part of the curve of infection. The period between October 2004 and February 2005 represents the inter-epidemic season. Asymptomatic excretion of HEV particles in the stools and the environmental reservoir of the HEV may explain the background noise observed and circulation of the virus.

Most patients came from the fourth and seventh districts of Bangui, but more cases of HEV infection were detected in patients living in the seventh and eighth districts. As the disease is related to exposure to faeces, hepatitis E is endemo-epidemic in areas where collective hygiene is badly monitored [3]. During epidemics, ingestion of viral particles by consumption of contaminated water is the principal mode of transmission, and more rarely by food soiled by human excreta. The seventh and eighth districts often have problems of overflowing drains and short-circuiting of networks of clean water and of structures to purify wastewater. Latrines are built close to wells, and water is usually used without treatment. All these insalubrities might explain the presence of the virus and the high rate of infection of the population in these two districts. Further, larger studies are necessary to determine the distribution of HEV in the districts of Bangui and to investigate the sources of the frequent outbreaks of HEV in this city since the first outbreak described in the suburbs in 2001 [25,26].

## Conclusion

This study shows that there are no specific clinical or physical symptoms that might allow clinicians to diagnose hepatitis E. The clinical signs, dominated by jaundice, vomiting, hepatalgia and asthenia, are not specific to HEV infection, but could orient the clinician in the event of an association with an evocative epidemiological context. In order to avoid repetitive outbreaks and epidemics of hepatitis E in Bangui, it would be desirable to reinforce the preventive measures of individual and collective hygiene and to set up a monitoring system for this pathology.

## Abbreviations

ALT: Alanine aminotransferase; AST: aspartate aminotransferase; CAR: Central African Republic; CDC: Centers for Disease Control and Prevention; HEV: hepatitis E virus; IgM: immunoglobulin class M; IgG: immunoglobulin class G; RNA: ribonucleic acid; RT-PCR: reverse transcriptase polymerase chain reaction assay; USA: United States of America; WHO: World Health Organization.

## Competing interests

The authors declare that they have no competing interests.

## Authors' contributions

AIG, an MD in training at Institut Pasteur de Bangui, participated in the design of the study, examined the patients and also participated in interpretation of the data; XK carried out the immunoassays; NPK conceived of and designed the study, directed its implementation, interpreted data and drafted the manuscript. All authors read and approved the final manuscript.

## Pre-publication history

The pre-publication history for this paper can be accessed here:

http://www.biomedcentral.com/1471-2334/11/93/prepub
